# The Glp-1 Analog Liraglutide Protects Against Angiotensin II and Pressure Overload-Induced Cardiac Hypertrophy via PI3K/Akt1 and AMPKa Signaling

**DOI:** 10.3389/fphar.2019.00537

**Published:** 2019-06-05

**Authors:** Ran Li, Yingguang Shan, Lu Gao, Xi Wang, Xule Wang, Fang Wang

**Affiliations:** ^1^Department of Cardiology, First Affiliated Hospital of Zhengzhou University, Zhengzhou, China; ^2^Department of Endocrinology, First Affiliated Hospital of Zhengzhou University, Zhengzhou, China

**Keywords:** liraglutide, cardiac hypertrophy, angiotensin II, AMPK a2, pressure overload

## Abstract

The Glp-1 analog, liraglutide (Lir), has been shown to reduce infarct size and improve cardiac function after myocardial ischemia in rodents with or without diabetes. However, the effect of Lir on angiotensin II (AngII) and pressure overload induced cardiac hypertrophy in nondiabetic mice and the underlying mechanisms are unclear. The aim of this study was to investigate the effect of Lir on cardiac hypertrophy induced by AngII infusion and pressure overload and to explore its possible mechanism. Mice were subjected to AngII as well as thoracic aorta coarctation (TAC) to induce a cardiac hypertrophy model. Mice were daily injected with either liraglutide or saline for 2 weeks after AngII infusion. Mice were also subjected to either liraglutide or saline for 25 days after TAC surgery. Neonatal rat cardiomyocytes and human AC cell lines were stimulated with AngII to induce a cardiomyocytes hypertrophy model. The results indicated Lir significantly inhibited cardiac hypertrophy and fibrosis and improved cardiac function in both the AngII and pressure overload induced model. The *in vitro* study showed that Lir inhibits AngII induced cell hypertrophy. Mechanistically, Lir directly suppressing the activation of PI3K/Akt1 and stimulated AMPKα signaling pathways in cardiomyocytes, which was confirmed by use of an mTOR activator (MHY1485), overexpression of constitutively active Akt, and the knockdown of AMPKa2 expression. Moreover, the protective effects of Lir were lost in AMPKa2 knockout mice. Taken together, Lir inhibits AngII and pressure overload induced cardiac remodeling via regulating PI3K/Akt1 and AMPKα signaling.

## Introduction

Cardiac hypertrophy is an independent risk factor contributing to the development of and mortality in cases of cardiovascular diseases (Ferrario, 2016). It may also occur as a complication associated with hypertension, valvular heart diseases, acute myocardial infarction, and congenital heart diseases (Ma et al., 2018). Pathological cardiac remodeling is initially invoked to reduce ventricular wall stress and temporally preserve cardiac pump function, eventually evolving into a devastating spiral of maladaptive alterations that culminate in HF and death (Wu et al., 2017). The signaling mechanisms involved in the remodeling response are complex, including phosphatidylinositol 3-kinase (PI3K)/protein kinase B (Akt), Ca^2+^/calcinerin, nuclear factor of activated T cells (NFAT), nuclear factor-kappa B (NF-κB), and mitogen-activated protein kinase (MAPKs) signaling. However, the molecular mechanisms have not been clearly elucidated, and its treatment is still facing a bottleneck. A better understanding of the factors that regulate remodeling pathways could reveal potential therapeutic targets for treating cardiac hypertrophy and heart failure.

Glucagon-likepeptide1 (GLP-1), an incretin hormone mainly derived from intestinal L-cells, stimulates insulin secretion, inhibits glucagon secretion, delays gastric emptying and reduces postprandial hyperglycemia (Dai et al., 2013; Kawatani et al., 2018). GLP-1 agonists and dipeptidyl peptidase-4 (DPP-4) inhibitors (that prevent the degradation of the GLP-1) have been approved for the treatment of type 2 diabetes (Trujillo and Nuffer, 2014). GLP1 and its analogs have also been demonstrated to exert cardioprotective actions in a few ischemia/reperfusion models (Ban et al., 2008; Noyan-Ashraf et al., 2009; Timmers et al., 2009). GLP1 analogs have been reported to reverse the molecular pathology and cardiovascular dysfunction in obese mice (Noyan-Ashraf et al., 2013). In 2009, Noyan-Ashraf et al. (2009) reported that the GLP-1R agonist liraglutide activates cytoprotective pathways and improves outcomes after experimental myocardial infarction in mice. Moreover, a Glp-1 analog, liraglutide, ameliorated hepatic steatosis, and cardiac hypertrophy in C57BL/6J mice fed a western diet (Mells et al., 2012). However, Kyhl et al. (2017) revealed that 4-week treatment with liraglutide did not affect cardiac remodeling following a nonreperfused myocardial infarction. These inconsistent results have caused confusion in regards to the usage of Lir in cardiovascular disease.

Although some of the studies have indicated GLP-1 agonists could reduce infarct size and improve cardiac function after myocardial ischemia in rodents with or without diabetes, the relative effects of these agents on cardiac remodeling in nondiabetic mice have not been studied. Furthermore, the underlying mechanisms of these agents in cardioprotection have not been completely delineated. Since Lir exerts cardioprotection in many other cardiovascular diseases, we assume Lir may be beneficial in cardiac hypertrophy, and thus, we used AngII and pressure loading to induce a cardiac remodeling model.

## Materials and Methods

### Animals and Animal Models

All of the studies were performed in accordance with the guidelines of the NIH (Guide for the Care and Use of Laboratory Animals, 1996) and were approved by the Animal Care and Use Committee of the First Affiliated Hospital of Zhenzhou University. A mouse cardiac hypertrophy model was induced by Ang II infusion. Ang II (1.4 mg/kg per day and dissolved in 0.9% NaCl) was subcutaneously infused for 4 weeks using an osmotic minipump (Alzet model 2004, Alza Corp.) implanted into each mouse. Saline-infused animals served as infusion controls and were subjected to the same procedures as the experimental animals, with the exception of the Ang II infusion. To investigate whether Lir can ameliorate cardiac hypertrophy, mice were randomized to receive intraperitoneal injections of Lir (200 μg/kg, dissolved in saline (0.9% NaCl), Novo Nordisk, Princeton, NJ, United States) or saline daily for 2 weeks after 2 weeks of AngII infusion when cardiac hypertrophy can be significantly documented. All of the mice (C57BL6J wild type mice) used in our studies were 8–10 week old males with a BW of 23.5–27.5 g, and the common diet for rodent intervention studies was used.

Adenosine 5^′^-monophosphate (AMP)-activated protein kinase (AMPKa)2 knockout mice (with a C57BL6J background) were purchased from Cyagen Biosciences Inc. (Guangzhou, China).

Thoracic aorta coarctation was performed as described in a previous study (Xiao et al., 2017). Mice were subjected to Lir (200 μg/kg, dissolved in saline, Novo Nordisk, Princeton, NJ, United States) from 3 days after TAC surgery until 28 days after surgery. The mice were sacrificed 4 weeks after TAC surgery.

After the mice were killed, their hearts were dissected and weighed to compare the heart weight/body weight (HW/BW, mg/g), heart weight/tibial length (HW/TL, mg/mm), and lung weight/body weight (LW/BW, mg/g) ratios.

### Echocardiographic Analyses and Pressure-Volume Loop

Mice were anesthetized with pentobarbital sodium (6 mg/kg, i.p.) for cardiac echocardiography using an ultrasound machine (Vivid 7, GE Medical System, Milwaukee, WI, United States). The left ventricular ejection fraction (LVEF), interventricular septal thickness at diastole (IVSD), LVESd, and LVEDd were calculated from the M-mode recording. Hemodynamics were measured in anesthetized (1.5% isoflurane) mice using cardiac catheterization. A microtip catheter transducer (SPR-839; Millar Instruments, Houston, TX, United States) was inserted into the right carotid artery and then advanced into the LV. Fifteen minutes after stabilization, pressure signals and the heart rate were continuously recorded with a Millar Pressure-Volume System (MPVS-400; Millar Instruments) coupled with a Powerlab/4SP A/D converter and then stored and displayed on a personal computer. Data were processed using the PVAN data analysis software.

### Quantitative Real-Time RT-PCR (RT-PCR)

Total RNA was extracted using the TRIzol reagent as previously described (Liu et al., 2015) and reverse transcribed. The first-strand cDNA was used for quantitative real-time PCR to quantify mRNA expression using GAPDH as the normalized control. The promoters used are listed in Table 1.

**Table 1:** Primer sequences used for RT-PCR.

mRNA	Forward	Reverse
ANP^a^	ACCTGCTAGACCACCTGGAG	CCTTGGCTGTTATCTTCGGTACCGG
BNP	GAGGTCACTCCTATCCTCTGG	GCCATTTCCTCCGACTTTTCTC
α-MHC	GTCCAAGTTCCGCAAGGT	AGGGTCTGCTGGAGAGGTTA
β-MHC^a^	CCGAGTCCCAGGTCAACAA	CTTCACGGGCACCCTTGGA
Col1agenI^a^	AGGCTTCAGTGGTTTGGATG	CACCAACAGCACCATCGTTA
Col1agenIII^a^	AAGGCTGCAAGATGGATGCT	GTGCTTACGTGGGACAGTCA
CTGF^a^	AGGGCCTCTTCTGCGATTTC	CTTTGGAAGGACTCACCGCT
TGFβ^a^	ATCCTGTCCAAACTAAGGCTCG	ACCTCTTTAGCATAGTAGTCCGC
GAPDH^a^	ACTCCACTCACGGCAAATTC	TCTCCATGGTGGTGAAGACA
ANP^b^	AAAGCAAACTGAGGGCTCTGCTCG	TTCGGTACCGGAAGCTGTTGCA
BNP^b^	CAGCAGCTTCTGCATCGTGGAT	TTCCTTAATCTGTCGCCGCTGG
β-MHC^b^	TCTGGACAGCTCCCCATTCT	CAAGGCTAACCTGGAGAAGATG
GAPDH^b^	GACATGCCGCCTGGAGAAAC	AGCCCAGGATGCCCTTTAGT
ANP^c^	CAGCAAGCAGTGGATTGCTCCT	TCTGCGTTGGACACGGCATTGT
BNP^c^	TGGAAACGTCCGGGTTACAGGA	TCCGGTCCATCTTCCTCCCAAA
β-MHC^c^	GGGCAAAGGCAAGGCCAAGAAA	ATGGGTGGAGCGCAAGTTGGTCA
GAPDH^c^	CATCACCATCTTCCAGGAGCGAGA	TGCAGGAGGCATTGCTGATGATCT


*Sequences are listed 5^′^–3^′^. ^*a*^The PCR used these primers for mice. ^*b*^The PCR used these primers for neonatal rat cardiomyocytes. ^*c*^The PCR used these primers in the AC cells.*

### Western Blot

Heart tissues and cardiomyocytes were lysed in RIPA buffer. Proteins were isolated as previously described (Liu et al., 2015). Briefly, the left ventricles were polished and the cardiomyocytes were lysed in RIPA buffer, and then, the protein concentrations were measured using the BCA Protein Assay Kit (Thermo, 23227) and an ELISA Reader (Synergy HT, Bio-Tek). The cell lysates (50 mg) were loaded into each lane and subjected to SDS-PAGE, and the proteins were then transferred onto Immobilon-FL membranes (Millipore, IPFL00010). The membranes were incubated overnight at 4°C with primary antibodies against one of the following proteins: phosphorylated (p-) and total (T) PI3K, Akt1, AMPKα, AMPKα, mammalian target of rapamycin (mTOR), ribosomal protein S6 kinase beta-1 (70S6K), S6, extracellular regulated protein kinases (ERK)1/2, NFAT2 and GAPDH (all of the antibodies were purchased form Cell Signaling Technology and diluted at 1:1000). The blots were scanned using a two-color infrared imaging system (Odyssey, LI-COR). Specific protein expression levels were normalized to the GAPDH protein for the total cell lysates and cytosolic proteins.

### Histological Analysis

Several heart sections (5 μm thick) were prepared as previously described (Liu et al., 2015). The cardiomyocyte histopathology was detected using HE. Single myocytes were measured using a quantitative digital image analysis system (Image-Pro Plus, version 6.0). Between 100 and 200 LV myocytes were outlined in each group. Collagen production was detected using PSR. Between 10 and 20 LV fields were outlined in each heart. All of the results were visualized and photographed via light microscopy (Eclipse8, Nikon, Tokyo, Japan) and the images were analyzed with ImagePro Plus software (Media Cybernetics, Bethesda, MD, United States) to quantify the cardiomyocyte area and the collagen percentage.

### Neonatal Rat Cardiomyocyte (NRCM) Culture

The 1- to 2-day-old Sprague-Dawley rats were killed by swift decapitation. Their hearts were quickly removed and only the ventricles were kept. They were washed with PBS three times and incubated with 0.125% trypsin-EDTA (Gibco, 2520-072) for 15 min. They were then enzymatically digested four times for 15 min in 0.125% trypsin-EDTA in PBS. Digestion was stopped by the addition of FBS at a final concentration of 10%. The cells were then centrifuged at 250 g for 8 min and then resuspended in DMEM/F12 (Gibco, C11330) supplemented with 10% FBS. The cells were then incubated for 1–2 h in 100-mm dishes to allow noncardiac myocytes (mainly cardiac fibroblasts) to adhere to the plastic. They were then plated in six well plates at a density of 5 × 10^5^ cells per well with 1% bromodeoxyuridine for 48 h. All of the antibodies used in the Western blotting are the same as those used in the *in vivo* studies. For immunofluorescence staining, an α-actin antibody (Abcam, diluted at 1:100) was used.

AC16 cells (ATCC, Bethesda, MD, United States) were cultured in DMEM supplemented with 12.5% fetal bovine serum and 1% penicillin-streptomycin. Cells in exponential growth were dissociated with 0.25% trypsin (GIBCO, 25200) and were seeded in six-well culture plates at a seeding density of 1 × 10^6^/well before being incubated for 24 h. Then, the cells were cultured with serum-free DMEM for another 12 h before treatment.

Cells were treated with Lir (25, 50, 100 nM) and AngII (1 μM) for 24 h. Cell viability was detected using CCK-8 assays. The expression levels of ANP, B-type natriuretic peptide (BNP), β-MHC, and α-MHC genes were detected by RT-PCR. The cardiomyocyte cross-sectional area in each group was evaluated by immunofluorescence staining for α-actin. After treatment with Ang II and/or Lir (25, 50, 100 nM) for 24 h, protein levels were detected by using WB assays. For cell transfection, replication-defective adenoviral (Ad) vectors were used to overexpress constitutively active Akt or to knock down AMPKa. The constitutively active Akt1 and NC adenoviral (Ad) vectors used in our study were generated by the Hanbio Biotechnology Co. (Shanghai, China). At 48 h after plating, the cardiomyocytes were infected with adenovirus diluted in DMEM/F12 at 100 MOI. After infection, cardiomyocyte cells were starved with FBS-free medium for 16 h and treated with Ang II and/or Lir for 24 h.

### Statistical Analysis

SPSS software, version 13.0, was used for data analysis. All data are expressed as the means ± SE. The Kolmogorov-Smirnov test was used to analyze the data distribution. All data were normally distributed. The groups were compared by one-way analysis of variance (ANOVA) followed by the *post hoc* LSD test when ANOVA found a significant value of F and no variance in homogeneity; otherwise, Tamhane’s T2 *post hoc* test was used. Comparisons between the two groups were performed using Student’s unpaired *t*-test. *p* < 0.05 indicates a statistical difference.

## Results

### Lir Attenuates the Development of Cardiac Hypertrophy and Dysfunction Induced by AngII

**FIGURE 1:**
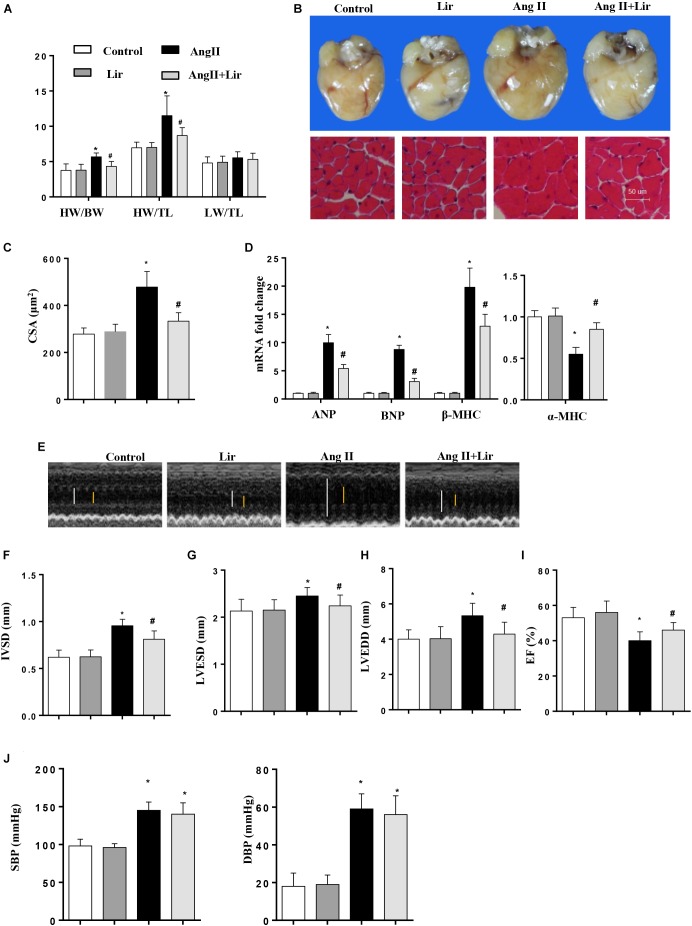
The effect of Lir on AngII-induced cardiac hypertrophy and dysfunction. Statistical results of the HW/BW, HW/TL, and LW/BW ratios (*n* = 10) at 4 weeks after AngII and/or Lir treatment **(A)**; the gross heart, HE staining **(B)** and CSA (*n* = 100 cells per section) **(C)** at 4 weeks after AngII and/or Lir treatment; RT-PCR analysis of hypertrophic markers including ANP, BNP, β-MHC, and α-MHC from the heart of different groups (*n* = 9) **(D)**; echocardiography results 4 weeks after AngII and/or Lir treatment (*n* = 10, white line: LVEDD, yellow line: LVESD) **(E–I)**. Pressure loop measurements of systolic (SBP) and diastolic blood pressure (DBP) in different groups (*n* = 10 mice/group, **J**). ^∗^*P* < 0.05, compared to the control group; ^#^*P* < 0.05, compared to the AngII group.

Mice were subjected to Ang II infusion to induce a cardiac hypertrophy model. Four weeks after the infusion, a significant hypertrophic response was observed as evidenced by an increased heart weight to body weight ratio (HW/BW), HW to tibial length (HW/TL) ratio, increased cross section area, increased transcription levels of hypertrophic markers [ANP, BNP, β-MHC] and a decreased transcription level of α-MHC. After 2 weeks of Lir treatment, these hypertrophic responses were lower than in the Ang II group as shown by decreased HW/BW and HW/TL ratios, a decreased cross section area, decreased transcription levels of hypertrophic markers (ANP, BNP, β-MHC) and an increased transcription level of α-MHC (Figure 1A–D).

We also detected the cardiac function by echocardiography. Four weeks after AngII infusion, the mice revealed ventricular wall hypertrophy and expansion as well as cardiac dysfunction as evidenced by an increased IVSd, LVEDd and LVESd, and decreased LVEF. Lir treatment ameliorated these changes with reduced IVSD, LVEDD and LVESD, and increases in LVEF compared with the Ang II group (Figure 1E–I). The systolic and diastolic blood pressure (SBP/DBP) were detected after 4 weeks of AngII infusion. SBP and DBP were found to be elevated in the AngII group, and Lir slightly reduced the SBP and DBP (*P* > 0.05) (Figure 1J). Collectively, Lir attenuates AngII induced cardiac hypertrophy and relieves cardiac dysfunction.

### Lir Attenuates Cardiac Fibrosis Induced by AngII *in vivo*

**FIGURE 2:**
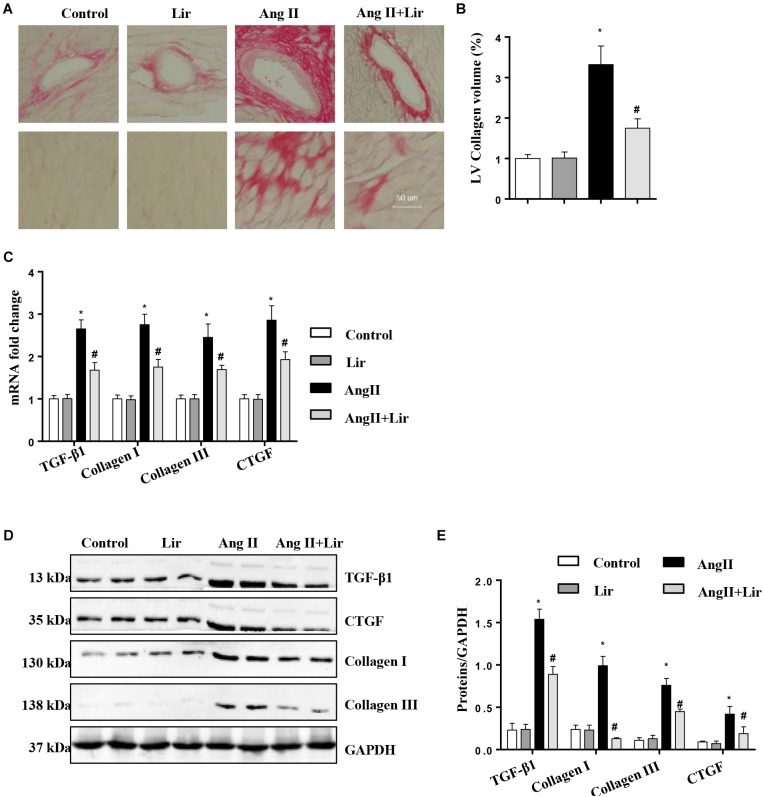
The effect of Lir on AngII-induced cardiac fibrosis. Histological sections of the heart were stained for PSR in the indicated groups at 4 weeks after AngII and/or Lir treatment. Left ventricular interstitial collagen volume fraction in the indicated groups was quantified using an image-analyzing system (**A,B**, the collagen in LV are shown in red); RT-PCR analysis of the mRNA expression of TGF-β1, collagen I, collagen III, and CTGF in the myocardium obtained from the indicated groups at 4 weeks after AngII and/or Lir treatment (*n* = 9) **(C)**. Western blot analysis of the expression of TGF-β1, CTGF, collagen I, and collagen III in the myocardium (*n* = 6) **(D,E)**. ^∗^*P* < 0.05, compared to the control group; ^#^*P* < 0.05, compared to the AngII group.

Cardiac fibrosis is one of the main features of cardiac hypertrophy. Thus, we determined the effects of Lir on cardiac fibrosis. PSR staining revealed a significant perivascular and interstitial fibrosis in the AngII infused mice heart while Lir attenuated this fibrosis level (Figure 2A,B). Subsequent analysis of mRNA and protein expression levels of TGF-β1, collagen I, collagen III, and CTGF, which are responsible for cardiac fibrosis, showed similar results (Figure 2C–E). These data suggested that Lir attenuated the cardiac fibrosis induced by Ang II.

### Lir Inhibits the PI3K/Akt Pathway and Promotes the Activation of AMPKα

**FIGURE 3:**
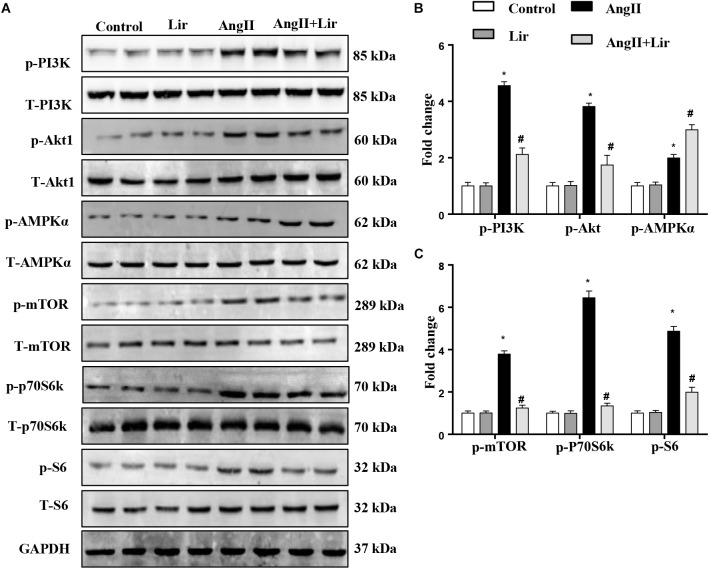
The effect of Lir on the AMPKα and Akt/mTOR/70S6K/S6 signaling pathways *in vivo*. The protein expression of phosphorylated and total PI3K, Akt, AMPKα, mTOR, 70S6K, and S6 in mice from the four groups. Representative WBs **(A)** and quantitative results **(B,C)** (*n* = 6). ^∗^*P* < 0.05, compared to the control group; ^#^*P* < 0.05, compared to the AngII group.

The molecular mechanism of the anti-hypertrophic effect of Lir was investigated. The main signaling pathway that contributes to cardiac hypertrophy was determined. We found that compared with the control mice, AngII-infusion significantly increased the phosphorylation level of PI3K as well as the phosphorylated level of downstream molecules Akt1, mTOR, p70S6K, and S6 (Figure 3A–C). We also detected the activation of AMPKa was increased in the AngII infused mice heart. After Lir treatment, the activated PI3K/Akt pathway was inhibited compared with that in the AngII group. Furthermore, the increased AMPKa activation induced by AngII was augmented after Lir treatment following AngII treatment (Figure 3A–C).

We also evaluated other hypertrophic signaling pathways such as ERK1/2 and NFAT. The levels of phosphorylated ERK and NFAT2 were increased in the AngII infused mice heart, but Lir did not affect the activation of ERK1/2 and NFAT2 (Supplementary Figures S1A,B). To detect whether the effects of liraglutide were mediated by activation of the GLP-1 receptor, the expression of GLP-1R was detected. As shown in Supplementary Figures S1C,D, GLP-1R expression was increased after stimulation with AngII but was not affected by liraglutide treatment in both NRCMs and ACs. These data suggest that Lir protects against cardiac hypertrophy through regulating PI3K/Akt1 and AMPKα.

### Lir Attenuates the Cardiomyocyte Hypertrophy Induced by AngII *in vitro*

**FIGURE 4:**
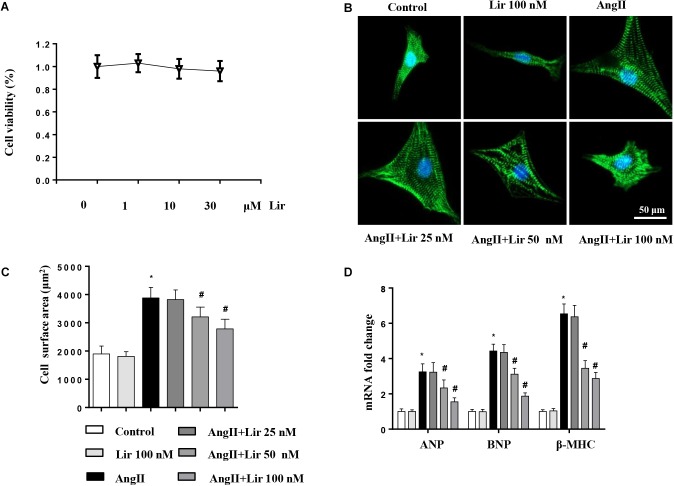
The effect of Lir on cardiomyocytes hypertrophy *in vitro*. Cardiomyocytes were treated with AngII and/or Lir (25, 50, 100 nM) for 24 h. **(A)** Cell viability of cardiomyocytes (*n* = 6). **(B)** Representative immunofluorescent staining of α-actin (*n* = 6). **(C)** Quantitative analysis of CSA (*n* > 50 cells per group). **(D)** RT-PCR analysis of the mRNA levels of ANP, BNP, and β-MHC in the indicated groups (*n* = 6). ^∗^*P* < 0.05, compared to the control group; ^#^*P* < 0.05, compared to the AngII group.

We then evaluated whether Lir directly affected cardiomyocytes. Cells were cultured and stimulated with Lir. The cell viability detected by the CCK-8 assay indicated that Lir (25, 50, 100 nM) did not affect cell viability compared with the control group (Figure 4A). AngII (1 μM) treatment induced significant cardiomyocytes hypertrophy as assessed by an increased CSA and increased transcription levels of hypertrophic markers (ANP, β-MHC) compared with that in the control group. However, 50 and 100 nM Lir but not 25 nM Lir greatly inhibited the cardiomyocytes hypertrophic response (a decreased CSA and reduced transcription levels of hypertrophic markers) (Figure 4B–D). These data indicated that Lir protects against cardiac hypertrophy via directly affecting cardiomyocytes.

Previous studies have reported that Lir decreased angiotensin type 1 receptor (AT1R) expression and increased angiotensin type 2 receptor (AT2R) expression, thus attenuating AngII-induced tissue fibrosis in rats (Zhang et al., 2015). Thus, we detected the expression patterns of AT1R and AT2R. As shown in Supplementary Figures S1E,F, AngII infusion increased AT1R expression and reduced AT2R expression but Lir did not affect the AT1R/AT2R expression pattern in both the AngII infused heart tissue and the cardiomyocytes. These data indicate that Lir exerts an anti-hypertrophy effect in mice that is not dependent on AT1R/AT2R.

### Lir Inhibits PI3K/Akt1 Signaling and Promotes AMPKα Activation *in vitro*

**FIGURE 5:**
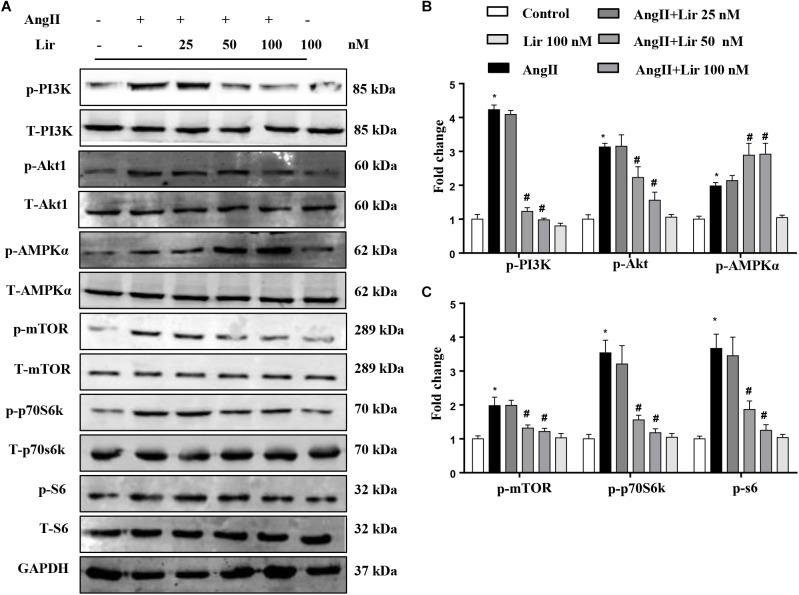
The effect of Lir on AMPKα and Akt/mTOR/70S6K/S6 signaling pathways in response to hypertrophic stimuli *in vitro*. The protein expression levels of phosphorylated and total PI3K, Akt, AMPKα, mTOR, 70S6K, and S6 in cardiomyocytes treated with AngII (1 μM) and Lir (1, 10, and 30 μM, respectively). Representative WBs **(A)** and quantitative results **(B,C)** (*n* = 6). ^∗^*P* < 0.05, compared to the control group; ^#^*P* < 0.05, compared to the AngII group.

To confirm the PI3K/Akt1 and AMPKa pathways mediated the protective effects of Lir in cardiomyocytes, these signaling pathways were studied *in vitro*. The activation of PI3K and its downstream targets Akt1, mTOR, p70S6K, and S6 were significantly activated in cardiomyocytes treated with Ang II (1 μM). However, 50 and 100 nM Lir reduced the activation levels of PI3K, Akt1, mTOR, p70S6K, and S6 (Figure 5A–C). We also examined AMPKa signaling. Consistent with the *in vivo* studies, AMPKa was activated after 30 min of Ang II stimulation, while 50 and 100 nM Lir further increased the activation of AMPKa (Figure 5A–C). These findings indicated that Lir attenuated cell hypertrophy predominantly through inhibiting the Akt1/mTOR signaling pathways and stimulating the AMPKα pathways in cardiomyocytes.

### Lir Attenuated the Hypertrophy of Cardiomyocytes in an Akt/AMPKα-Dependent Manner *in vitro*

**FIGURE 6:**
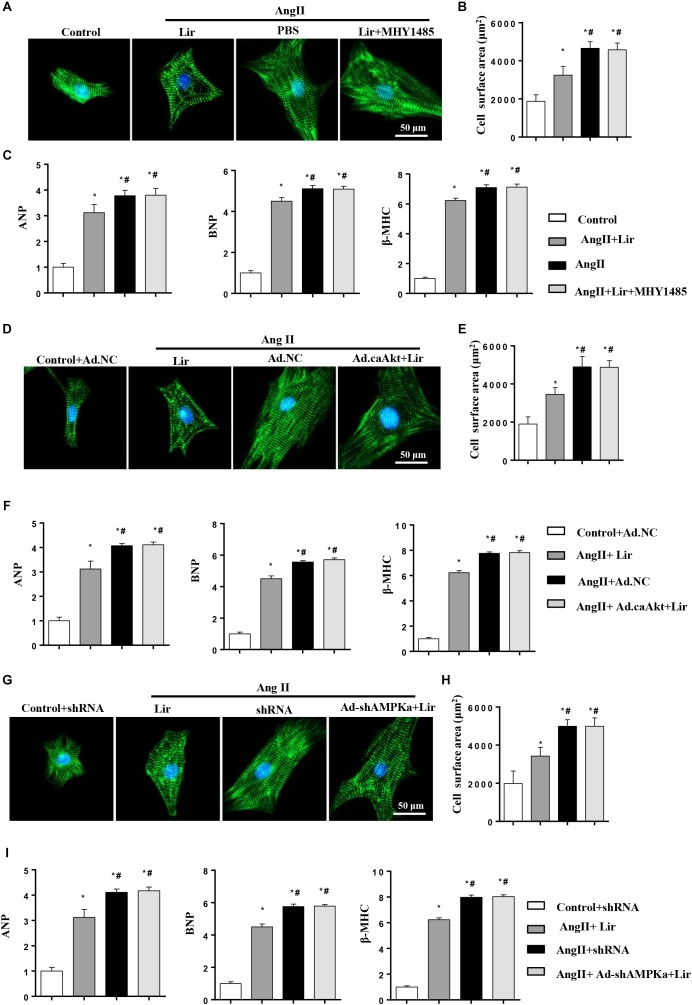
Lir attenuated hypertrophy of myocytes in an AMPKα-dependent manner *in vitro*. Cardiomyocytes were treated with AngII (1 μM) and Lir (30 μM) with/without MHY1485 (10 μM), Ad.caAkt or Ad-shAMPKa infection. **(A,D,G)** Representative immunofluorescent staining of α-actin in the indicated group (*n* = 6). **(B,E,H)** Quantitative analysis of CSA in the indicated group (*n* = 50 + cells per group). **(C,F,I)** RT-PCR analysis of the mRNA levels of ANP, BNP, and β-MHC in the indicated group (*n* = 6). ^∗^*P* < 0.05, compared to the control group **(B,C)**/Control + Ad.NC **(E,F)**/Control + shRNA **(H,I)**; ^#^*P* < 0.05, compared to the AngII group **(B,C)**/AngII + Ad.NC **(E,F)**/AngII + shRNA **(H,I)**.

To decipher the effects of Lir on PI3K/Akt1/mTOR and AMPKa signaling, cardiomyocytes were subjected to an mTOR activator (MHY1485, 10 μM, MCE China) or infected with constitutive active Akt1 (Ad.caAkt) to activate Akt1 or Ad-shAMPKa to knockdown AMPKa expression. We found the anti-hypertrophic effect of Lir on cardiomyocytes was abolished by MHY1485, Ad.caAkt, and shAMPKa since the CSA and mRNA expression of hypertrophic markers showed no significant differences among the AngII + Lir + MHY1485/Ad.caAkt/Ad-shAMPKa groups and the AngII group but were increased relative to the AngII + Lir group (Figure 6A–I). These data suggest that both activation of Akt and inhibition of AMPKa could counteract the anti-hypertrophy of Lir *in vitro*.

### AMPKa Deficiency Abolished the Anti-hypertrophic Effects of Lir *in vivo*

**FIGURE 7:**
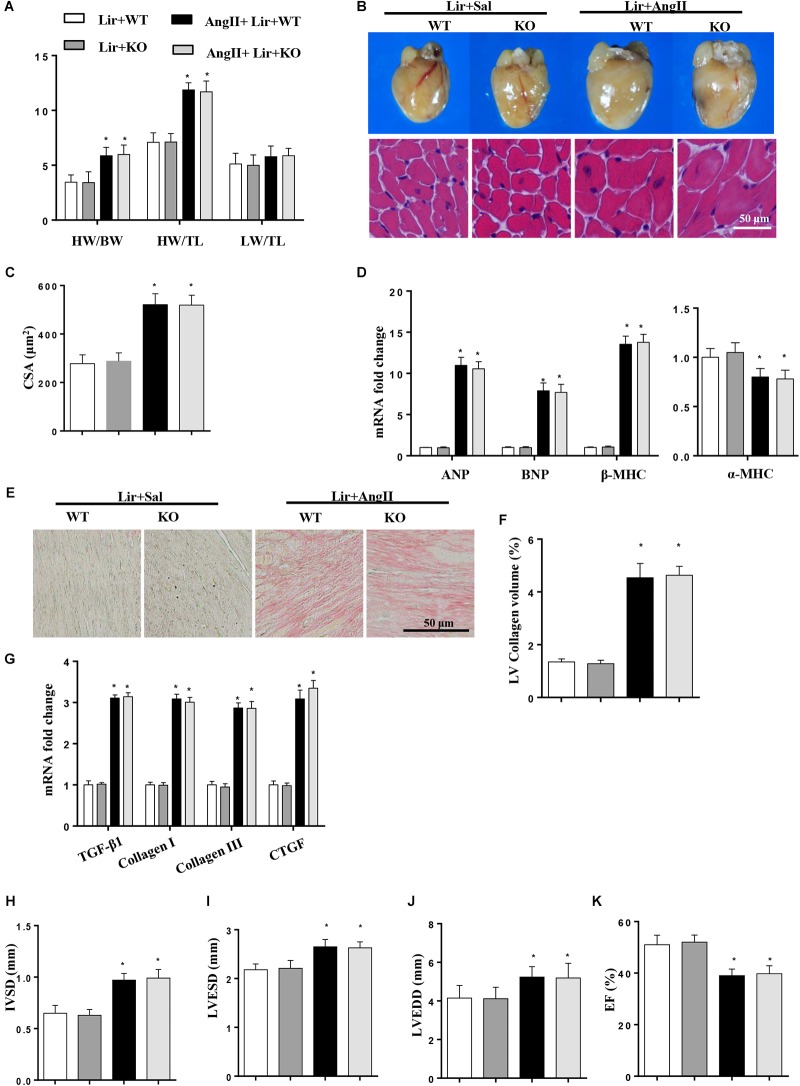
AMPKa deficiency abolished the anti-hypertrophic effects of Lir *in vivo*. AMPKa knockout mice were injected with Lir 2 weeks after Ang II administration. Statistical results of the HW/BW, HW/TL, and LW/BW ratios (*n* = 10) at 4 weeks after AngII and Lir treatment **(A)**; the gross heart, HE staining **(B)** and CSA (*n* = 100 cells per section) **(C)** at 4 weeks after AngII infusion; RT-PCR analysis of hypertrophic markers including ANP, BNP, β-MHC, and α-MHC from the heart of different groups (*n* = 9) **(D)**; Left ventricular interstitial collagen volume fraction in the indicated groups **(E,F)**; RT-PCR analysis of the mRNA expression of TGF-β1, collagen I, collagen III, and CTGF in the myocardium obtained from the indicated groups (*n* = 9) **(G)**; echocardiography results in the indicated group (**H–K**, *n* = 10). ^∗^*P* < 0.05, compared to the Lir-WT group; ^#^*P* < 0.05, compared to the AngII + Lir-WT group.

To further investigate whether Lir exerted protective effects through AMPKα *in vivo*, AMPKa knockout mice were injected with Lir after 2 weeks of AngII infusion. Consistent with the results *in vitro*, AMPKa knock-out counteracted the protective effects of Lir and was characterized by the same extent of HW/BW, HW/TL, LW to BW ratios, CSA, LV collagen volume, hypertrophic and fibrotic markers transcription levels and the same extent of cardiac dysfunction between Lir treatment in wild type mice and Lir treatment in knockout mice (Figure 7A–K).

### The Effect of Lir on Human Cardiomyocytes Hypertrophy

**FIGURE 8:**
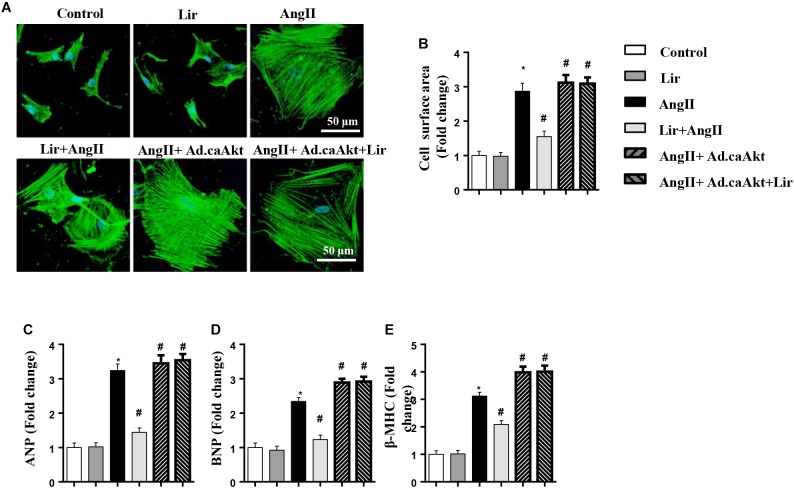
The effect of Lir on human cardiomyocytes. AC16 cells were stimulated with Ang II (1 μM) in the presence of Lir (100 nM) and infected with Ad.caAkt. **(A)** Representative immunofluorescent staining of α-actin (*n* = 6). **(B)** Quantitative analysis of CSA (*n* > 50 cells per group). **(C–E)** RT-PCR analysis of the mRNA levels of ANP, BNP, and β-MHC in the indicated groups (*n* = 6). ^∗^*P* < 0.05, compared to the control group; ^#^*P* < 0.05, compared to the AngII group.

To confirm the anti-hypertrophy effect of Lir on human cardiomyocytes, AC16 cells were stimulated with Ang II (1 μM) in the presence of Lir (100 nM) and infected with Ad.caAkt to express activated Akt1. Consistent with the results in rat cardiomyocytes, Lir inhibits Ang II induced AC16 cell hypertrophy (a reduced CSA and reduced transcription of ANP and β-MHC) and Ad.caAkt abolished these protective effects of Lir as assessed by the increased CSA and transcription of ANP and β-MHC in the AngII + Ad.caAkt + Lir group compared with that in the AngII group and the AngII + Lir group (Figure 8A–E).

### The Effect of Lir on Pressure Overload Induced Cardiac Hypertrophy in Mice

**FIGURE 9:**
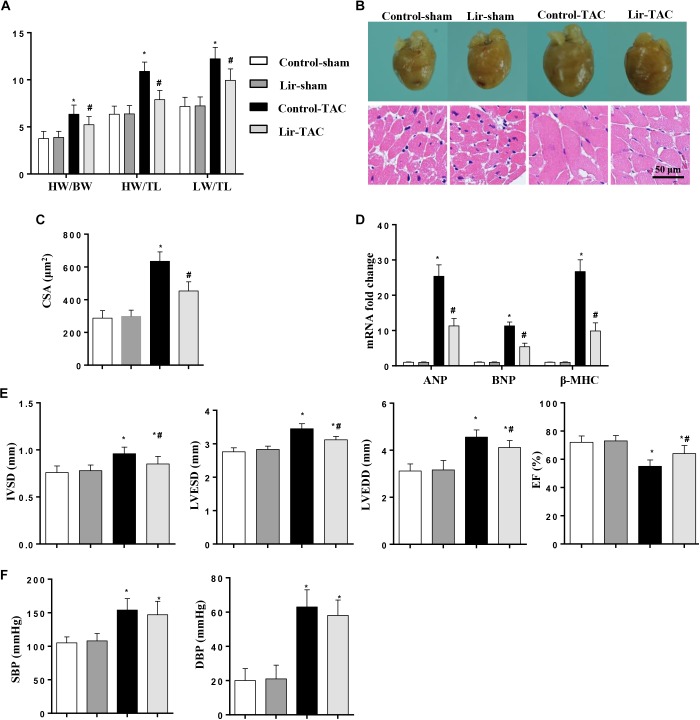
The effect of Lir on pressure overload induced cardiac hypertrophy. Mice were subjected to TAC surgery and treated with Lir for 25 days. Statistical results of the HW/BW, HW/TL, and LW/BW ratios (*n* = 10) at 4 weeks after TAC **(A)**. The gross heart, HE staining **(B)** and CSA (*n* = 100 cells per section) **(C)** at 4 weeks after TAC. RT-PCR analysis of hypertrophic markers including ANP, BNP, and β-MHC from the heart of the different groups (*n* = 9) **(D)**. Echocardiography results at 4 weeks after TAC (**E**, *n* = 10). Pressure loop measurements of systolic (SBP) and diastolic blood pressure (DBP) in different groups (*n* = 10 mice/group, **F**). ^∗^*P* < 0.05, compared to the corresponding sham group; ^#^*P* < 0.05, compared to the control-TAC group.

To confirm the effect of Lir on the other cardiac hypertrophy model, mice were subjected to TAC surgery and treated with Lir for 25 days. After 4 weeks of TAC, HW/BW, HW/TL, and LW/TL were remarkably increased in the control-TAC group. The CSA and the transcription levels of the hypertrophic markers were also increased in the control-TAC group (Figure 9A–D). Lir treatment attenuated the extent of the cardiac hypertrophy as assessed by decreased HW/BW, HW/TL, LW/TL, CSA, and reduced transcription levels of these hypertrophic markers (Figure 9A–D). Lir also improved the cardiac dysfunction induced by pressure overload (Figure 9E). The systolic and diastolic blood pressure (SBP/DBP) were measured 4 weeks after TAC surgery. SBP and DBP were found to be elevated in the TAC group and Lir slightly reduced the SBP and DBP (*P* > 0.05) (Figure 9F).

## Discussion

Cardiac remodeling is an important adaptive response of cardiomyocytes to a variety of mechanical insults such as pressure overload and neurohormonal stimuli such as Ang II, endothelin-1, and adrenaline, which eventually lead to heart failure (Wu J. et al., 2014). It is known that pressure overload can activate the renin-angiotensin system (RAS) and induced the release of AngII, which activates the Galpha(q) protein-coupled receptor (GPCR) signaling pathway (Wu J. et al., 2014; Wu et al., 2015). Thus, we used both Ang II stimuli and a pressure overload model for *in vivo* study to induce cardiac remodeling in mice. In this study, we demonstrated Lir could attenuate AngII and pressure overload-induced cardiac remodeling, improving the cardiac dysfunction. Lir had a direct effect on the cardiomyocytes that inhibited the AngII-induced cardiomyocytes hypertrophic response. These protective effects were mediated by inhibition of the PI3K/Akt1 pathway and activation of AMPKa. We found that both continuous activation of Akt1 and knock down of AMPKa could abrogate the protective effect of Lir. Our study has revealed a novel approach to the treatment of cardiac remodeling.

The PI3K/Akt pathway is one of the key signaling cascades activated upon IGF-1 receptor (IGF-1R) stimulation. This pathway plays a central role in regulating metabolism, glucose uptake, proliferation and protein synthesis, all of which have the single goal of promoting cell survival (Wu et al., 2017). Mammals express three isoforms of Akt: Akt1, Akt2, and Akt3. Akt1-KO mice show a reduced cardiac muscle mass and display defects in physiological growth (Chen et al., 2001). When subjected to pressure overload, these mice develop an exacerbated form of cardiac hypertrophy (DeBosch et al., 2006). Among the downstream effectors of Akt, mTOR is activated by Akt phosphorylation. mTORC1, a complex form of mTOR, stimulates protein synthesis through the eukaryotic initiation factor 4E-BP1 and p70S6 kinase during protein translation, and the acceleration of protein translation can enhance cell growth and mass (Aoyagi and Matsui, 2011; Wu Q.Q. et al., 2014). A previous study reported that Lir exerted cardioprotection via mTOR/ULK1-dependent autophagy in glucose toxicity-induced cardiac injury (Yu et al., 2018). Lir has also been reported to regulate PI3K/Akt signaling in type 2 diabetic rats (Yang et al., 2018; Yu et al., 2018). In this study, we found that PI3K/Akt1 signaling was activated after 4 weeks of AngII infusion. Lir inhibits PI3K/Akt1 signaling. Activation of mTOR or Akt1 could abolish the protective effects of Lir on cardiomyocyte hypertrophy. These findings suggest that Lir exerted an anti-hypertrophic effect by inhibition of PI3K/Akt signaling.

AMP-activated protein kinase is a sensor of energy status. When the cellular AMP/ATP ratio increases, AMPK is activated by phosphorylation, leading to reduced energy consumption, and increased energy production by the cells (Viollet et al., 2003). AMPK deletion mice display dilated cardiomyopathy and cardiac dysfunction in the absence of pathological stress (Sung et al., 2015), which proves the importance of AMPK in the cardiovascular physiological process. Studies have shown the benefits of many AMPKα activators in protection against LV remodeling (Zong et al., 2013; Xu et al., 2018). Thus, increasing the activation of AMPKa may be a therapeutic target for cardiac hypertrophy. In the heart, insulin stimulates a variety of kinase cascades and controls glucose utilization. Insulin is able to activate Akt and inactivate AMPK in the heart (Kovacic et al., 2003). Studies have reported that Akt and AMPKa are closely associated with each other. Akt activity negatively regulates the phosphorylation of AMPK in cardiomyocytes (Kovacic et al., 2003). Lir was reported to exert neuroprotective effects via activation of AMPKa signaling in a diabetic model (Kong et al., 2018; Yang et al., 2018). In this study, AMPKα activity was increased in AngII stimulated cardiomyocytes. Lir intervention further augmented the activation of AMPKa in cardiomyocytes. AMPKa knockout abolished the protective effects of Lir *in vivo*. These data indicate that AMPKa signaling regulation also mediated the protective effects of Lir on cardiac hypertrophy. The crosstalk of Akt and AMPKa signaling in cardiomyocytes contributes to the downregulation of the hypertrophic response. Thus, merely knocking out AMPKa or activation of Akt could totally counteract Lir’s cardio-protection effects.

For further confirmation, the effect of Lir on cardiomyocyte hypertrophy was observed *in vitro* in both neonatal rat cardiomyocytes and human AC16 cardiomyocytes following AngII treatment. The results showed that Lir reduced the AngII-induced hypertrophic response in both neonatal rat cardiomyocytes and human AC16 cardiomyocytes. Furthermore, activation of Akt in both neonatal rat cardiomyocytes and human AC16 cardiomyocytes counteracted Lir’s cardio-protection effects, which is consistent with the *in vivo* results. These results suggest promising results from application of Lir to treat cardiac hypertrophy patients in clinical practice.

As indicated by a large number of researchers, myocardial fibrosis is associated with cardiac hypertrophy, myocardial infarction, hypertension, and heart failure (Daniel et al., 2016; Shimizu and Minamino, 2016; Wu et al., 2016). Myocardial fibrosis is a common pathological change during the development of various heart diseases. A major presentation of ventricular remodeling, myocardial fibrosis can lead to myocardial stiffness, impaired ventricular diastolic function, decreased coronary flow reserve, and even sudden death (Porter and Turner, 2009; Sirish et al., 2013). Lir was reported to exert anti-renal fibrosis via inhibition of the epithelial-mesenchymal transition (Li et al., 2018). Lir improves myocardial fibrosis after myocardial infarction through inhibition of CTGF by activating cAMP in mice (Huang et al., 2018). Our findings provide valuable experimental data for clinical research on the protective effect of Lir. Thus, it is important to explore the mechanisms that stimulate collagen deposition in the heart and define approaches to limit these processes. As shown here, Lir treatment significantly attenuated cardiac fibrosis after AngII-infusion. These results showed that Lir can inhibit the fibrosis induced by AngII-infusion. The mechanism underlying the anti-fibrosis effect of Lir on cardiac hypertrophy may attribute to the cardio-protection of Lir, which exerts a beneficial communication between cardiomyocytes and fibroblasts. Further study into the anti-fibrosis effect of Lir based on fibroblast experiments is still needed.

As a GLP-1R agonist, whether the antihypertrophic action of Lir is directly mediated by GLP-1R is unclear. Our data suggest that after stimulation with AngII, the expression of GLP-1R was elevated, but Lir did not affect the expression level of GLP-1R. Thus, these data provide indirect evidence that the effects of Lir on AMPKα signaling do not rely on GLP-1R. However, whether GLP-1R mediates the effects needs to be elucidated.

Studies have also reported an anti-hypertension effect of Lir (Rondinelli et al., 2017; Simonds et al., 2019). In our study, we found that Lir treatment could slightly reduce the SBP and DBP induced by both AngII infusion and TAC surgery (without a significant difference). This inconsistency may be attributed to the differences in dosage used and the treatment duration. Overall, in our study the anti-hypertrophy effect of Lir is irrelevant to blood pressure reduction. Since we did not design a group with Ang II-infused/TAC surgery animals sacrificed 2 weeks after the infusion/surgery, a limitation of our study exists in that whether the anti-hypertrophic and anti-fibrotic effects seen with liraglutide treatment were reversing Ang II-induced effects or were only halting the effects of Ang II is unknown. Further studies are needed to clarify this question.

## Conclusion

In conclusion, our present study showed for the first time that Lir can ameliorate cardiac hypertrophy by regulating the PI3K/Akt and AMPKa signaling pathways. The advantage of Lir is that Lir is currently in clinical use for treatment of type 2 diabetes. This gives Lir an advantage over many other small molecular peptides, plant extracts, or even gene therapies that are currently undergoing exploration to treat cardiac hypertrophy. Further studies of the comparison of Lir with other clinical anti-hypertrophic agents such as beta receptor blockers, angiotensin converting enzyme inhibitors, and angiotensin II receptor antagonists are needed.

## Ethics Statement

All of the studies were performed in accordance with the guidelines of the NIH (Guide for the Care and Use of Laboratory Animals, 1996) and were approved by the Animal Care and Use Committee of the First Affiliated Hospital of Zhenzhou University.

## Author Contributions

RL and FW contributed to the conception and designed the experiments. RL, YS, and LG carried out the experiments. XiW and XuW analyzed the experimental results and revised the manuscript. RL and FW wrote and revised the manuscript.

## Conflict of Interest Statement

The authors declare that the research was conducted in the absence of any commercial or financial relationships that could be construed as a potential conflict of interest.

## ABBREVIATIONS

AMPK: AMP-activated protein kinase
AngII: angiotensin II
ANP: atrial natriuretic peptide
BNP: brain natriuretic peptide
BW: body weight
CSA: cell surface area
CTGF: connective tissue growth factor
EF: ejection fraction
FS: fractional shortening
HE: hematoxylin and eosin
HW: heart weight
IVSd: interventricular septal end diastolic dimension
Lir: lirentin
LV: left ventricular
LVEDd: left ventricular end-diastolic dimension
LVESd: left ventricular end-systolic dimension
LW: lung weight
NC: negative control
PSR: picroSirius red
TAC: thoracic aorta coarctation
TGF-β1: transforming growth factor β1
TL: tibia length
α-MHC: α-myosin heavy chain
β-MHC: β-myosin heavy chain
