# Supplementary material for: The Glp-1 Analog Liraglutide Protects Against Angiotensin II and Pressure Overload-Induced Cardiac Hypertrophy via PI3K/Akt1 and AMPKa Signaling
Source: Front Pharmacol. 2019 Jun 5;10:537. doi: 10.3389/fphar.2019.00537 (PMC6560159; doi:10.3389/fphar.2019.00537)
Supplement: Supplementary file 1 [file Image_1.pdf]

Figure S1

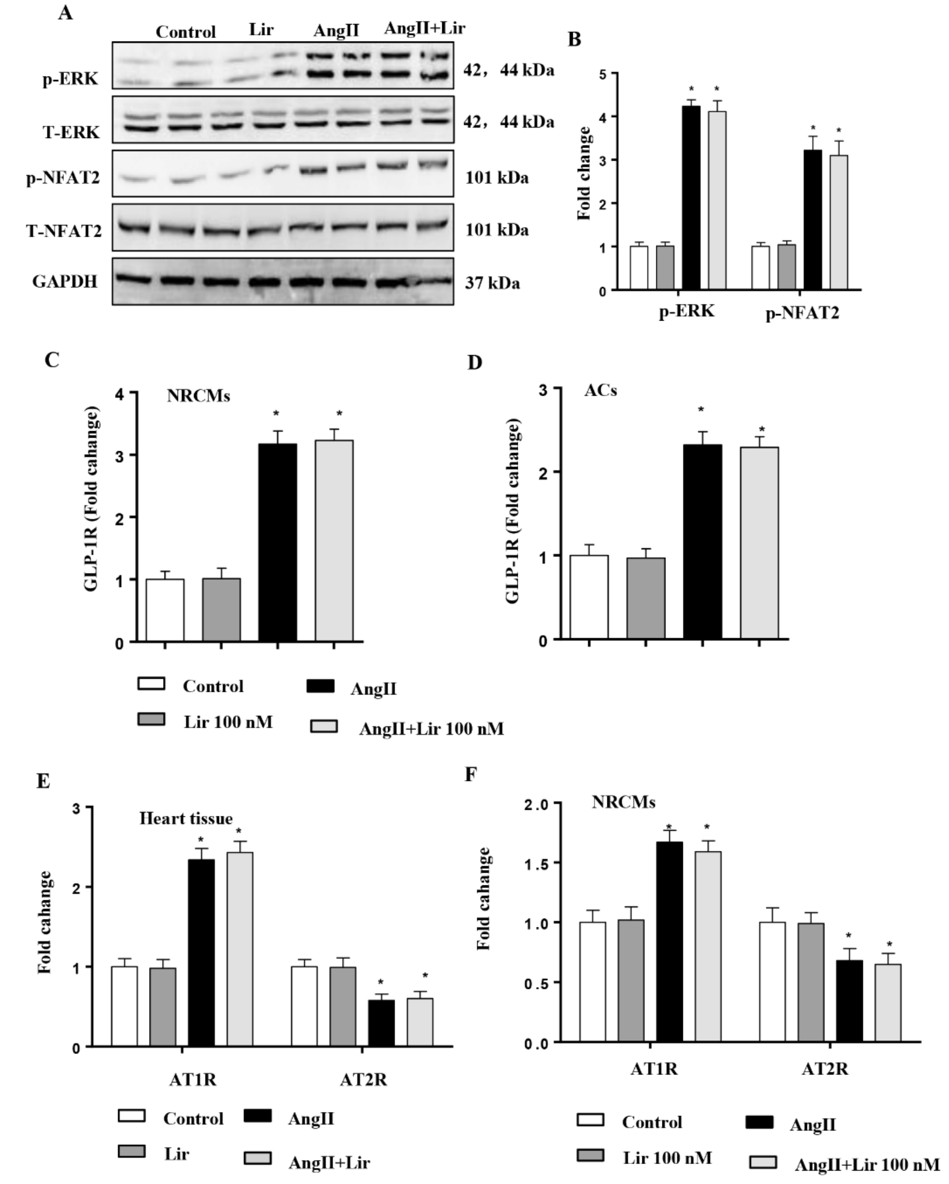

Figure S1. The effect of Lir on ERK and NFAT2 signalling

The protein expression of phosphorylated and total ERK and NFAT2 in mice from the four groups. Representative WB (A) and quantitative results (B) (n=6). *P＜0.05, compared to control group. C and D: The expression level of GLP-1R level in NRCMs and ACs after stimulated with Ang II and treated with liraglutide (100nM). *P＜0.05, compared to control group. E: The mRNA expression level of AT1R and AT2R in heart tissue with AngII infusion. F: The mRNA expression level of AT1R and AT2R in

NRCMs after AngII stimulation. *P＜0.05, compared to control group.
